# Physical Activity, Guideline Compliance, and Exercise Preferences in Axial Spondyloarthritis: A Comparative Cross-Sectional Study

**DOI:** 10.3390/jcm15176706

**Published:** 2026-08-29

**Authors:** Serpil Demirulus, Gamze Kilic, Murat Karkucak, Erhan Capkin

**Affiliations:** 1 Department of Physical Medicine and Rehabilitation, Yavuz Selim Bone Diseases and Rehabilitation Hospital, Trabzon 61030, Turkey; 2 Division of Rheumatology, Department of Physical and Rehabilitation Medicine, Hacettepe University Medical School, Ankara 06230, Turkey; gkilic.md@hotmail.com; 3 Department of Physical and Rehabilitation Medicine, Karadeniz Technical University School of Medicine, Trabzon 61080, Turkey; muratkarkucak@yahoo.com (M.K.); drcapkin@yahoo.com (E.C.)

**Keywords:** axial spondyloarthritis, physical activity, exercise, compliance

## Abstract

**Background:** Exercise is a cornerstone of axial spondyloarthritis (axSpA) management, yet real-world adherence to physical activity (PA) recommendations and patient preferences for exercise delivery remain insufficiently characterized. This study aimed to compare PA levels, World Health Organization (WHO) aerobic PA guideline compliance, and exercise preferences between patients with axSpA and age and sex-matched healthy controls. **Methods:** A total of 153 patients with axSpA and 153 age- and sex-matched healthy controls were recruited from a tertiary rheumatology clinic. PA was assessed using the International Physical Activity Questionnaire-Short Form. Compliance with WHO aerobic PA recommendations was defined as achieving ≥150 min/week of moderate-intensity PA including walking, ≥75 min/week of vigorous-intensity PA, or an equivalent combination. Disease activity, function, quality of life, kinesiophobia, fibromyalgia, neuropathic pain, psychological status, and exercise preferences (adapted from the Personalized Exercise Questionnaire) were also recorded, and logistic regression identified factors associated with compliance among axSpA patients. **Results:** Total IPAQ-SF MET-min/week scores were comparable between groups, but vigorous-intensity activity was lower in axSpA (*p* = 0.012). Fewer patients with axSpA met WHO aerobic PA recommendations than controls (60.1% vs. 71.2%, *p* = 0.041) and this gap was most evident among women (43.4% vs. 75.5%, *p* = 0.002) but not among men, with a significant axSpA status × sex interaction (*p* = 0.008). Among patients with axSpA, female sex and higher kinesiophobia scores were independently associated with lower odds of meeting the WHO aerobic PA recommendations. Patients with axSpA also showed distinct exercise preferences including greater preference for health-professional supervision, home-based exercise, and regular feedback. **Conclusions:** Patients with axSpA had lower adherence to WHO aerobic PA recommendations than healthy controls. Among patients with axSpA, female sex and higher kinesiophobia were associated with lower adherence. These findings highlight the importance of considering sex-related differences, kinesiophobia, and individual exercise preferences when developing personalized strategies to promote PA in axSpA.

## 1. Introduction

Axial spondyloarthritis (axSpA) is a chronic inflammatory rheumatic disease characterized by inflammatory back pain, morning stiffness, impaired spinal mobility, and limitations in physical function [1]. As symptoms often begin in early adulthood, reduced physical activity (PA) may have long-term consequences for functional capacity, social participation, cardiometabolic health, and quality of life. Beyond axial symptoms, fatigue, sleep disturbance, psychological distress, and impaired quality of life contribute to the multidimensional burden of the disease [2,3,4,5]. In this context, PA and exercise are not merely supportive lifestyle measures, but rather essential components of long-term axSpA management [6].

Physical activity encompasses all bodily movement that increases energy expenditure, whereas exercise represents a structured, purposeful and repetitive form of PA aimed at improving or maintaining fitness [7]. The current World Health Organization (WHO) guidelines recommend that adults engage in at least 150–300 min of moderate-intensity PA or 75–150 min of vigorous-intensity PA, or an equivalent combination of both per week [8]. The 2021 European Alliance of Associations for Rheumatology (EULAR) recommendations further state that the WHO healthy lifestyle recommendations are applicable to people with rheumatic and musculoskeletal diseases, including those with inflammatory arthritis and axSpA [9]. In addition, contemporary axSpA management recommendations emphasize regular PA and exercise as core components of standard care, alongside pharmacological treatment [6,9].

Beyond overall activity volume, the specific types of PA that patients with axSpA engage in also matter for both efficacy and safety. Across observational studies from different countries, walking and stretching are among the most frequently reported activities, while swimming or aquatic exercise and cycling are also commonly undertaken [10,11,12]. Aquatic exercise has been rated by patients as providing greater symptom relief and enjoyment than other exercise modalities [10]. In addition, structured high-intensity endurance and resistance exercise has randomized controlled pilot-trial evidence suggesting beneficial effects on disease activity and cardiovascular risk factors [13]. Aerobic exercise is generally encouraged in axSpA, with the type and intensity adapted according to disease stage, structural involvement, physical fitness and individual safety considerations. Expert recommendations also emphasise the importance of spinal mobility, flexibility, strengthening, and postural education in exercise programmes [14,15].

Not all PA is equally beneficial, and some forms may carry increased risk, particularly in patients with more advanced structural changes. An evidence-based consensus statement on exercise in ankylosing spondylitis (AS) states that high-impact activities require case-by-case assessment and may be contraindicated in advanced disease. Activities that substantially challenge balance, postural stability, or cardiorespiratory capacity should likewise be assessed individually [15]. This caution reflects the vulnerability of the rigid, ankylosed spine to highly unstable fractures, even after low-energy trauma, with the cervical spine being the most frequently affected region [16]. These considerations highlight the importance of PA counselling in axSpA, which should focus not only on achieving sufficient activity volume but also on providing guidance toward effective and safe activity types that are appropriately matched to the individual’s disease stage.

Despite clear recommendations supporting regular and appropriately tailored PA, the extent to which patients with axSpA achieve the recommended PA levels in daily life remains variable. Previous studies have reported considerable variability in adherence to WHO PA recommendations among patients with axSpA, with approximately one-half to three-quarters of patients meeting recommended activity levels across different cohorts [17,18,19,20]. This variability suggests that adherence is influenced not only by disease-related factors, but also by broader patient-reported, psychological, and contextual determinants. Pain, stiffness, fatigue, perceived exertion, and lack of time have been identified as common barriers to PA participation in this population [11,12,21,22].

Although previous studies have examined PA levels, guideline adherence, and their clinical correlates in axSpA [17,18,19,20], important scientific and clinical gaps remain. However, relatively limited evidence has simultaneously evaluated adherence to aerobic PA recommendations, the clinical and patient-reported factors associated with adherence, and patients’ exercise preferences, including preferred activity type, setting, timing, and professional support. Integrating these domains within a comparative framework may provide a more comprehensive and clinically relevant understanding of PA behaviour in axSpA and may help translate general exercise recommendations into individualized counselling strategies.

Therefore, this study aimed to compare adherence to WHO aerobic PA recommendations between patients with axSpA and healthy controls, identify clinical and patient-reported determinants of adherence, and characterize exercise preferences regarding activity type, setting, timing, and professional support. By integrating PA behavior with patient preferences within a comparative framework, this study may provide clinically relevant information to support individualized exercise counselling and facilitate the implementation of PA recommendations in routine clinical practice.

## 2. Materials and Methods

### 2.1. Study Design and Participants

This comparative cross-sectional study was conducted at a tertiary rheumatology outpatient clinic. The study protocol was approved by the Ethical Committee of the Karadeniz Technical University Medical Faculty (Protocol number: 2024/145) and all participants provided written informed consent prior to enrollment. The patients were enrolled between August 2024 and September 2025.

Consecutive adult patients (aged ≥18 years) who met the Assessment of SpondyloArthritis International Society (ASAS) classification criteria for axSpA [23] were recruited. Healthy controls were frequency-matched to the axSpA group according to sex and 5-year age strata. Matching was performed at the group level to achieve comparable age and sex distributions between the groups. Participants were excluded if they had any condition that could substantially impair participation in PA or interfere with the completion of study assessments, including neurological, orthopedic, cardiopulmonary, or systemic conditions; a history of major surgery or significant trauma within the past six months; current pregnancy; severe psychiatric disorders; or cognitive impairments. For the healthy control group, individuals with any diagnosed inflammatory rheumatic diseases, chronic musculoskeletal pain syndromes, or other systemic pathologies capable of altering physical activity behaviors were excluded.

### 2.2. Sociodemographic and Clinical Assessments

Sociodemographic and clinical characteristics of all participants, including age, sex, and body mass index (BMI), were recorded. Sleep quality and fatigue severity were evaluated using validated patient-reported outcome measures. Sleep quality was assessed via the Pittsburgh Sleep Quality Index (PSQI); global scores greater than 5 indicate poor sleep quality, reflecting significant difficulties in sleep initiation, maintenance, and overall satisfaction [24]. Fatigue severity was measured using the Fatigue Severity Scale (FSS) [25]. The Turkish versions of both the PSQI and FSS have been shown to be valid and reliable [26,27].

### 2.3. Physical Activity Assessment

Physical activity was assessed using the International Physical Activity Questionnaire-Short Form (IPAQ-SF), a 7-item questionnaire that assesses time spent walking, sitting, and engaging in moderate- and vigorous-intensity PA during the previous week. PA was analyzed as continuous scores expressed in metabolic equivalent task (MET)-minutes per week. One MET represents an energy expenditure of approximately 1 kcal/kg/hour at rest [28]. Based on responses obtained from the IPAQ-SF, minutes per week of moderate PA, including walking, and vigorous PA were calculated for each participant. Walking minutes were added to moderate-intensity activity minutes to obtain the total weekly moderate-intensity PA duration, whereas vigorous-intensity activity was calculated separately [29]. Compliance with the WHO aerobic PA recommendations was defined as achieving ≥150 min/week of moderate-intensity PA including walking, ≥75 min/week of vigorous-intensity PA, or an equivalent combination calculated as moderate-intensity minutes + 2 × vigorous-intensity minutes [8,29]. Participants were then dichotomized according to whether they met these recommendations. The Turkish version of the IPAQ-SF has been shown to be valid and reliable [30].

### 2.4. Exercise Preferences and Perceived Barriers

Exercise preferences and perceived barriers were assessed using a brief, study-specific questionnaire adapted from previously published research (Personalized Exercise Questionnaire (PEQ)) [31]. Items 1, 2, 19, 20, 22, 25–28, 34, and 35 of the original PEQ were selected to assess preferred exercise settings, timing, perceived barriers, and support needs. These items were used for exploratory descriptive purposes rather than as a validated psychometric scale; therefore, no total score was calculated, and the findings were interpreted descriptively. The content and wording of the questionnaire were reviewed by the authors to ensure clinical relevance, clarity, and comprehensibility for patients with axSpA (Supplementary Materials S1). Participants were additionally asked to indicate their preferred types of exercise, with multiple selections permitted.

### 2.5. Disease-Specific Assessments

Disease activity was evaluated using the Bath Ankylosing Spondylitis Disease Activity Index (BASDAI) [32] and the Ankylosing Spondylitis Disease Activity Score incorporating C-reactive protein (ASDAS-CRP) [33]. Spinal mobility was assessed via the Bath Ankylosing Spondylitis Metrology Index (BASMI) [34], while physical function was quantified using the Bath Ankylosing Spondylitis Functional Index (BASFI) [35] and the Health Assessment Questionnaire for Spondyloarthritis (HAQ-S) [36]. Additionally, overall functioning and health status were measured using the Assessment of SpondyloArthritis international Society Health Index (ASAS-HI) [37], and health-related quality of life was evaluated via the Ankylosing Spondylitis Quality of Life (ASQoL) questionnaire [38]. Higher BASDAI and ASDAS-CRP scores indicate greater disease activity; higher BASMI scores indicate greater spinal mobility limitation; higher BASFI and HAQ-S scores indicate greater functional limitation or disability; higher ASAS-HI scores indicate worse overall health and functioning; and higher ASQoL scores indicate poorer disease-specific quality of life [32,33,34,35,36,37,38]. The Turkish versions of BASDAI, BASFI, HAQ-S, ASAS-HI, and ASQoL were used, all of which have been shown to be valid and reliable [37,39,40,41,42].

### 2.6. Other Clinical Assessments

Psychological status, including symptoms of anxiety and depression, was assessed using the Hospital Anxiety and Depression Scale (HADS) [43]. Fibromyalgia syndrome (FMS) was screened using the Fibromyalgia Syndrome Rapid Screening Test (FIRST) [44]. Neuropathic pain symptoms were evaluated using the Douleur Neuropathique en 4 Questions (DN4) questionnaire [45]. Kinesiophobia, defined as the fear of movement due to the belief that movement may cause pain or injury, was evaluated using the Tampa Scale for Kinesiophobia (TSK) [46]. Higher TSK scores indicate greater levels of kinesiophobia. Validated and reliable Turkish versions of the HADS, FIRST, DN4, and TSK were used [47,48,49,50].

### 2.7. Statistical Analysis

Statistical analyses were performed using IBM SPSS Statistics version 27.0 (IBM Corp., Armonk, NY, USA). The normality of continuous variables was assessed visually (histograms and Q-Q plots) and with the Shapiro–Wilk test. Continuous variables with a non-normal distribution are presented as median and interquartile range (IQR), whereas normally distributed variables are presented as mean ± standard deviation (SD). Categorical variables are expressed as frequencies and percentages. Because matching was performed at the group level rather than by individual pairing, the axSpA and control groups were treated as independent samples in the statistical analyses. Comparisons between the axSpA and control groups were performed using the Mann–Whitney U test for non-normally distributed continuous variables and the independent-samples t-test for normally distributed variables. Categorical variables were compared using the Pearson chi-square test or Fisher’s exact test when appropriate.

Exercise preference items derived from the PEQ were analyzed using the Pearson chi-square test or Fisher’s exact test, as appropriate. To account for multiple comparisons, the Benjamini–Hochberg false discovery rate (FDR) procedure was applied separately to each PEQ-derived item and its corresponding set of response-category comparisons. Both unadjusted and FDR-adjusted *p*-values are reported.

Adherence to the WHO aerobic PA recommendations was assessed using the IPAQ-SF. Participants were classified as meeting the aerobic PA recommendations if they reported at least 150 min of moderate-intensity PA per week, at least 75 min of vigorous-intensity PA per week, or an equivalent combination of moderate- and vigorous-intensity PA. A binary outcome variable was generated, with 0 indicating that the participant did not meet the recommendations and 1 indicating that the participant did.

In the primary analysis, reported walking minutes were incorporated into the assessment of adherence as moderate-intensity PA. As a sensitivity analysis, walking minutes were excluded from the calculation of adherence, and the outcome was recalculated using only reported moderate- and vigorous-intensity PA. Multivariable models were further adjusted for age, sex, and BMI.

Variables considered clinically relevant and/or associated with the outcome at a univariate *p*-value < 0.20 were included in the multivariable binary logistic regression model, following the purposeful variable selection approach described by Bursac et al. [51]. Multicollinearity was assessed using variance inflation factor (VIF) and tolerance statistics. Odds ratios (ORs) with 95% confidence intervals (CIs) were calculated to identify independent factors associated with meeting the WHO aerobic PA recommendations.

Model performance was evaluated using the Omnibus test of model coefficients, Nagelkerke’s R^2^, the Hosmer–Lemeshow goodness-of-fit test, and overall classification accuracy. Effect modification by sex was assessed by including a disease group × sex interaction term in the multivariable logistic regression model.

All statistical tests were two-tailed, and a *p*-value < 0.05 was considered statistically significant. For analyses involving multiple comparisons, statistical significance was interpreted according to the Benjamini–Hochberg FDR-adjusted *p*-values.

## 3. Results

A total of 306 participants were included in this study: 153 patients with axSpA and 153 age- and sex-matched healthy controls. Regarding sleep quality, the axSpA group had significantly higher PSQI total scores (*p* < 0.001), and poor sleep quality (PSQI > 5) was markedly more frequent among them (*p* < 0.001). Fatigue severity was also greater in the axSpA group (*p* = 0.003), with a significantly higher proportion of participants experiencing clinically relevant fatigue (FSS ≥ 4) than controls (63.4% vs. 49.0%, *p* = 0.011). The sociodemographic and clinical characteristics of the participants are shown in Table 1.

International Physical Activity Questionnaire-Short Form results are presented in Table 2. Median vigorous PA was significantly lower in the axSpA group compared with controls (*p* = 0.012), whereas no significant differences were observed for moderate-intensity PA, walking, or total PA between the groups (all *p* > 0.05). Although overall PA levels did not differ significantly between groups, a significantly smaller proportion of axSpA patients met the aerobic PA guideline recommendations compared with controls (60.1% vs. 71.2%, *p* = 0.041).

**Figure 1:**
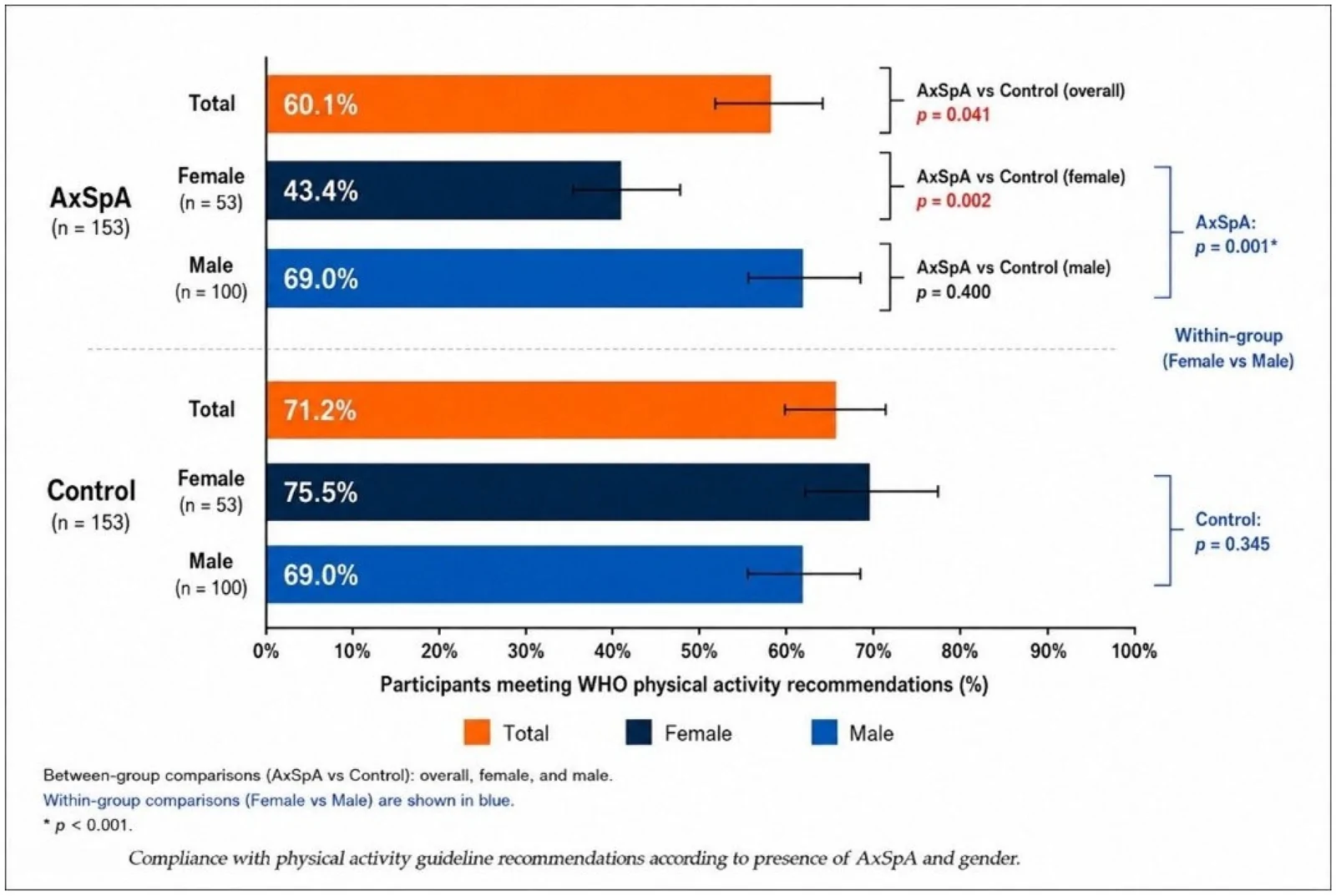
The rate of participants meeting the WHO aerobic PA recommendations by disease group and sex. The disease group × sex interaction was statistically significant (*p* = 0.008).

Compliance with WHO aerobic PA recommendations differed between groups and by sex (Figure 1). In the primary analysis, compliance was significantly lower in the axSpA group compared with controls (60.1% vs. 71.2%). Among female participants, compliance was markedly lower in axSpA patients than in controls (43.4% vs. 75.5%, *p* = 0.002), whereas compliance rates among male participants were comparable between groups (69.0% vs. 69.0%, *p* = 0.400). A formal interaction analysis demonstrated a significant interaction between the axSpA group and sex (*p* = 0.008), indicating that the association between axSpA and meeting the WHO aerobic PA recommendations differed by sex. In a sensitivity analysis excluding walking from the calculation, 24.8% of patients with axSpA and 36.6% of controls met the WHO aerobic PA recommendations (*p* = 0.026).

**Figure 2:**
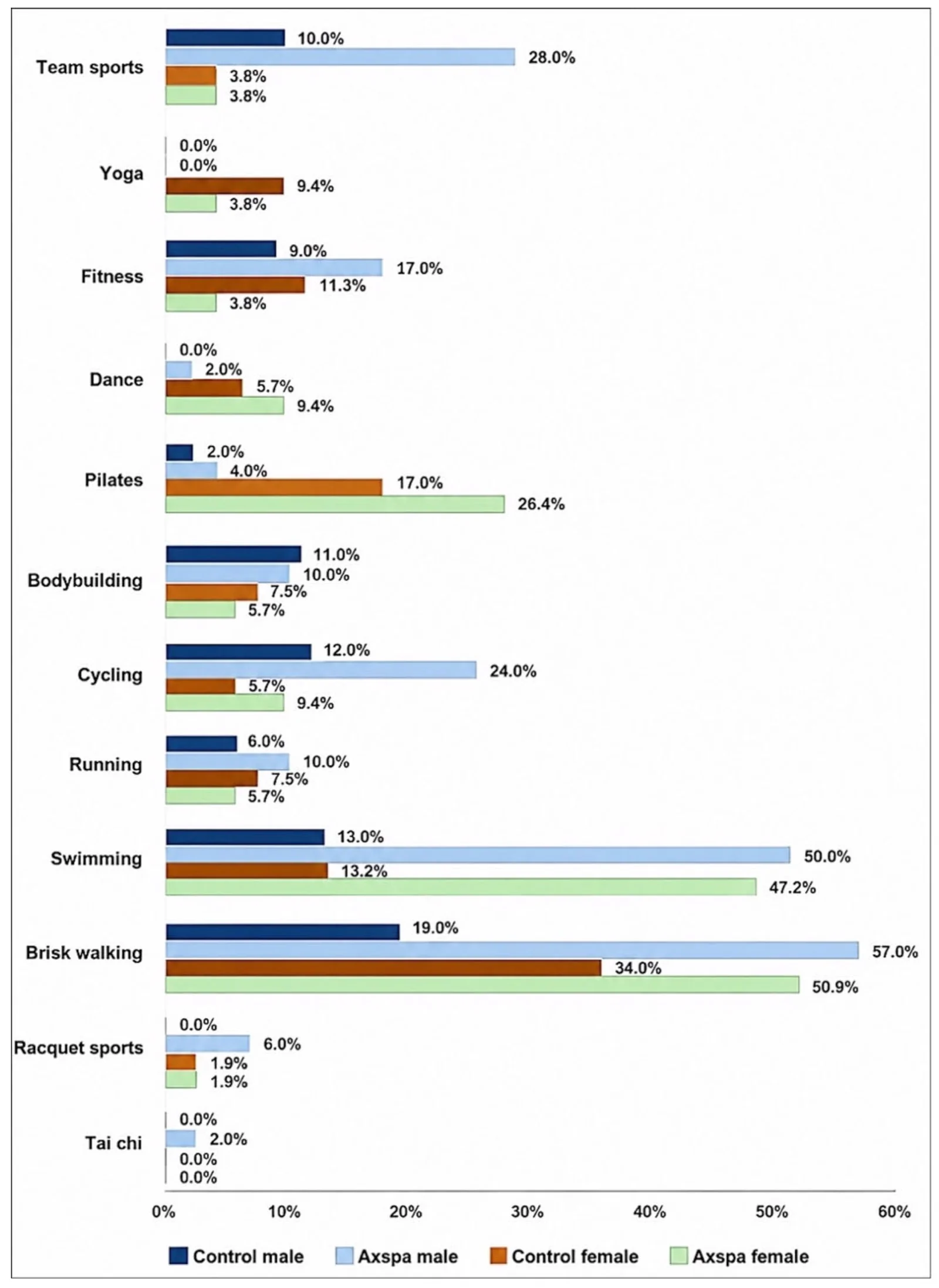
Preferred exercise types among patients with axial spondyloarthritis and healthy controls.

Exercise preferences differed by group and sex (Figure 2). Brisk walking was the most commonly preferred activity across all subgroups, particularly among patients with axSpA of both sexes. Swimming was also frequently preferred, with similarly high preference rates among male and female patients with axSpA. Cycling and team sports were more commonly preferred by male participants, especially in the axSpA group, whereas Pilates, yoga, and dance were predominantly preferred by female participants, with higher frequencies observed among axSpA females compared with healthy controls. Preferences for activities such as running, bodybuilding, racquet sports, and tai chi was generally low across all groups.

Clinically relevant variables with a univariate *p* value < 0.20 were included in a multivariable binary logistic regression model (Supplementary Table S1). No missing data was present. Due to multicollinearity, BASDAI, BASMI, ASQoL, and HADS-A score variables were excluded. The overall model was statistically significant (*p* < 0.001) and explained 28.3% of the variance in meeting the WHO aerobic PA recommendations (Nagelkerke R^2^ = 0.283). Model calibration was adequate according to the Hosmer–Lemeshow goodness-of-fit test (χ^2^ = 3.213, df = 8, *p* = 0.920). The model correctly classified 79.1% of participants, with a sensitivity of 34.2% and specificity of 93.9%.

After adjustment for covariates, female sex (OR = 0.274, 95% CI: 0.090–0.835, *p* = 0.023) and higher TSK scores (OR = 0.838, 95% CI: 0.750–0.937, *p* = 0.002) were independently associated with meeting the WHO aerobic PA recommendations. Age, FSS, BASFI, HAQ-S, PSQI, and ASAS-HI were not independently associated with meeting the recommendations (Table 3).

In the corresponding adjusted sensitivity analysis after excluding walking, axSpA remained associated with lower odds of meeting the recommendations (OR = 0.568, 95% CI: 0.339–0.951, *p* = 0.032; Supplementary Table S2).

Responses to items adapted from the PEQ are given in Supplementary Table S3. Overall, significant between-group differences were observed in several exercise preferences. Compared with healthy controls, participants with AxSpA were more likely to prefer supervision by a healthcare professional, home-based exercise, receiving regular feedback regarding exercise progress, and telephone-based follow-up. Conversely, healthy controls more frequently preferred exercising in a gym, participating in small-group exercise sessions, and receiving feedback via e-mail. After Benjamini–Hochberg correction, the significant differences regarding supervision by healthcare professionals, gym versus home preference, small-group exercise, telephone feedback, e-mail feedback, mobility limitations, finances, sleep quality, and confidence remained statistically significant.

## 4. Discussion

This study evaluated PA levels, compliance with global guideline recommendations, and exercise preferences among patients with axSpA compared with age- and sex-matched healthy controls. The primary findings indicated that although total IPAQ-SF MET-min/week scores were comparable between the two groups, patients with axSpA were significantly less likely to meet the WHO aerobic PA recommendations. This gap in compliance was particularly pronounced among female patients with axSpA. Among patients with axSpA, female sex and higher kinesiophobia were independently associated with lower adherence to the WHO aerobic PA recommendations.

Despite the widely recognized role of exercise as a cornerstone in the management of axSpA, there remains a notable discrepancy between clinical recommendations and the actual PA levels observed in these patients [12,17,18,20,52]. In the present study, 60.1% of patients with axSpA met the WHO PA recommendations, which is broadly consistent with the results of other research groups that calculated compliance from total weekly minutes using the same WHO thresholds. In a study conducted in France, 54.7% of patients with axSpA were found to comply with WHO recommendations [12]. In the Dutch GLAS and LUMC cohorts, 72–77% of patients met the aerobic component of the WHO guidelines [17]. A study conducted in Singapore reported a compliance rate of 77%, although its equivalent-combination criterion was defined as a 600 MET-minute threshold rather than a direct minute-based calculation [20]. Taken together, these variations likely reflect differences in sociocultural contexts and disease characteristics, as well as methodological differences in how compliance was operationalized across instruments.

The discrepancy between comparable total IPAQ-SF MET-min/week scores and lower WHO guideline adherence in patients with axSpA reflects the distinction between overall PA volume and attainment of specific duration and intensity thresholds. Total PA scores may be substantially influenced by walking and lower-intensity activities, while meeting WHO aerobic PA guideline recommendations requires reaching a defined duration and intensity threshold. Comparable total MET-min/week values, therefore, do not necessarily reflect comparable adherence to guideline recommendations. The lower level of vigorous-intensity activity observed in the axSpA group may partly account for their lower compliance rate, despite an overall PA volume similar to that of controls. However, the observed between-group difference should be interpreted cautiously in light of potential confounding.

To date, evidence exploring the clinical and patient-reported determinants of PA guideline compliance in axSpA remains remarkably limited, as most prior research has predominantly focused on overall PA levels. Among the scarce data available, studies have generally suggested that age, sex, functional status, disease activity, disease duration, and anti-TNF therapy use do not independently influence whether a patient meets WHO recommendations in patients with axSpA [12]. Instead, active physical therapy engagement has been identified as the primary driver of guideline adherence [52]. In contrast, our findings showed that female sex and higher TSK scores were independently associated with lower odds of meeting the WHO aerobic PA recommendations. The significant axSpA status × sex interaction further indicated that the association between axSpA status and adherence differed by sex, supporting the greater between-group disparity observed among women. The association between higher kinesiophobia and lower adherence to PA recommendations also suggests that kinesiophobia may be relevant to sufficient PA behaviour in axSpA. This outcome aligns with the existing evidence that identifies kinesiophobia as a significant factor in reduced PA participation in axSpA [53], and is consistent with the data indicating a link between higher kinesiophobia and lower PA in non-radiographic axSpA patients [21]. Accordingly, these findings suggest that considering both sex differences and kinesiophobia may represent a valuable approach when trying to promote PA among this population.

A pivotal finding of this study was that patients with axSpA demonstrated a significantly higher preference for supervised exercise under the guidance of healthcare professionals compared to controls, who more commonly favored personal trainers. This distinction likely reflects the patients’ perception of exercise as a therapeutic intervention rather than a performance-oriented activity, underscoring the critical role of professionally supervised exercise programs in the management of axSpA. This behavioral preference is supported by current clinical studies, which establish supervised exercise as an important component of non-pharmacological management that offers superior benefits over unsupervised routines [54,55]. Additionally, professionally monitored programs achieve moderate to high effect sizes in reducing disease activity while significantly improving physical function and spinal mobility [55]. Qualitative evidence further reinforces this by highlighting that supervision is a key factor in enhancing patient adherence to PA [56].

Regarding exercise modalities, brisk walking and swimming were the most commonly preferred activities in both groups, with particularly high preference frequencies among individuals with axSpA, consistent with the findings of Fabre et al. [12] who similarly identified these as the predominant exercise choices in this population. From a biomechanical perspective, these low-impact activities are ideally tolerated in axSpA as they minimize axial loading and mechanical stress on the spine and sacroiliac joints while maintaining cardiorespiratory fitness. Notably, sex-specific exercise preferences were observed, with Pilates being more frequently preferred by women with axSpA. In contrast, healthy controls more frequently preferred activities such as team sports and cycling.

Although descriptive differences in preferred exercise timing were observed between the groups, these differences did not remain statistically significant after FDR adjustment. In contrast, differences in exercise-related feedback preferences were more consistent. Patients with axSpA were more likely to prefer providing feedback on their exercise programs and more frequently favored direct communication methods, particularly in-person and telephone-based communication, whereas e-mail was more commonly preferred by controls. These findings suggest that patients with axSpA may favor more direct and interactive forms of communication regarding their exercise programs, which may be relevant when designing individualized exercise support strategies.

Concerning perceived barriers, axSpA patients reported significantly greater challenges across multiple domains, including mobility limitations, sleep disturbances, financial constraints, and reduced self-confidence. These findings align with the prior literature demonstrating that symptom burden, fatigue, and psychosocial factors are key determinants of physical activity participation in this population [21,57,58]. These findings suggest that both physical and psychosocial barriers should be considered when developing individualized PA support strategies.

Collectively, these findings underscore that exercise promotion in axSpA cannot be approached through a one-size-fits-all framework. Rather, individualized prescription that integrates patient preferences, sex-related differences, and perceived multidimensional barriers may help improve engagement with aerobic PA guideline recommendations.

The main strength of this study lies in its multidimensional assessment of PA and exercise-related behaviors in patients with axSpA. In addition to evaluating adherence to WHO-recommended aerobic PA guidelines, we comprehensively explored exercise preferences, including exercise type, setting, timing, feedback expectations, and perceived barriers. By comparing these factors with those of healthy controls, this study provides a broader understanding of PA adherence, exercise preferences, and perceived barriers. However, several limitations of this study should be acknowledged. First, its cross-sectional design precludes causal inference and limits the assessment of longitudinal changes in PA and exercise preferences. Second, PA and exercise preferences were assessed using self-reported questionnaires, which may be subject to recall and reporting bias, and objective measures such as accelerometry were not available. In addition, the exercise preference questionnaire was adapted for exploratory purposes and was not a validated psychometric instrument. Third, BMI differed significantly between the axSpA and control groups and residual confounding cannot be excluded. Finally, this was a single-center study conducted in a cohort with relatively modest disease activity and a high proportion of patients receiving biologic therapy, particularly anti-TNF agents, which may limit the generalizability of the findings. Moreover, only the aerobic component of the WHO PA recommendations was assessed, and muscle-strengthening activity was not evaluated.

Taken together, patients with axSpA had lower compliance with PA recommendations than healthy controls. Among patients with axSpA, female sex and higher kinesiophobia were associated with lower odds of adherence. Compared with healthy individuals, patients with axSpA preferred supervised and home-based exercise, reported different exercise preferences, and faced different patterns of barriers to maintaining regular PA. These findings underscore the importance of considering sex-related differences, kinesiophobia, and patient preferences when developing personalized strategies to encourage PA among patients with axSpA.

## Figures and Tables

**Table 1:** Demographic and clinical characteristics of participants.

	AxSpA *n* = 153	Control *n* = 153	*p*
Sex (male)	100 (65.4)	100 (65.4)	1.00
Age (years)	40 (34–47)	40 (33–47)	0.877
BMI (kg/m^2^)	26.8 (24.2–30.4)	25.1 (23.4–27.5)	<0.001
PSQI	6 (3–9)	2 (1–4)	<0.001
PSQI (>5)	81 (52.9)	23 (15.0)	<0.001
FSS	4.7 (3.1–5.9)	3.9 (3.1–5.0)	0.003
FSS (≥4)	97 (63.4)	75 (49.0)	0.011
Disease-specific characteristics			
Time since diagnosis (years)	9 (3–13)	N/A	
Medical treatment for AxSpA		N/A	
None	10 (6.5)		
Regular NSAIDs at full dose	13 (8.5)		
Irregular NSAIDs use	5 (3.3)		
Sulfasalazine	4 (2.6)		
Anti-TNF agents	115 (75.2)		
IL-17A inhibitors	3 (2.0)		
JAK inhibitors	3 (2.0)		
BASDAI	2.6 (1.2–4.2)	N/A	
ASDAS-CRP	2.4 (1.6–3.2)	N/A	
BASMI	2.1 (1.2–3.2)	N/A	
BASFI	1.0 (0.2–2.8)	N/A	
HADS-A	6 (2–9)	N/A	
HADS-D	5 (2–9)	N/A	
HAQ-S	0.3 (0.1–0.8)	N/A	
ASQoL	5 (2–10)	N/A	
ASAS-HI	6 (3–9)	N/A	
FIRST	2 (0–4)	N/A	
DN4	2 (0–4)	N/A	
TSK	40.5 ± 5.1	N/A	

Chi-square or Mann–Whitney U tests were used. Data are presented as *n* (%) or mean ± SD/median (IQR). ASAS-HI: Assessment of Spondylarthritis international Society Health Index; ASDAS-CRP: Ankylosing Spondylitis Disease Activity Score with C-reactive protein; ASQoL: Ankylosing Spondylitis Quality of Life; BASDAI: Bath Ankylosing Spondylitis Disease Activity Index; BASFI: Bath Ankylosing Spondylitis Functional Index; BASMI: Bath Ankylosing Spondylitis Metrology Index; BMI: Body Mass Index; DN4: Douleur Neuropathique 4; FIRST: Fibromyalgia Rapid Screening Tool; FSS: Fatigue Severity Scale; HADS-A/D: Hospital Anxiety and Depression Scale-Anxiety/Depression; HAQ-S: Health Assessment Questionnaire for Spondyloarthropathies; IL-17A: Interleukin 17A, N/A: Non-applicable, NSAID: Non-steroidal anti-inflammatory drug; PSQI: Pittsburgh Sleep Quality Index; TNF: Tumor necrosis factor, TSK: Tampa Scale for Kinesiophobia.

**Table 2:** International Physical Activity Questionnaire-Short Form (IPAQ-SF) results (MET-min/week), and comparisons between the AxSpa and control groups.

	AxSpA *n* = 153	Control *n* = 153	*p*
Vigorous activity	0 (0–0)	0 (0–480)	0.012
Moderate activity	0 (0–240)	0 (0–320)	0.139
Walking	445.5 (198–792)	495 (132–792)	0.615
Total PA	693 (247.5–1653)	872 (396–1695)	0.383
Meeting the WHO aerobic PA recommendations	92 (60.1)	109 (71.2)	0.041

Chi-square or Mann–Whitney U tests were used. Data are presented as *n* (%) or mean ± SD/median (IQR). MET: Metabolic Equivalent of Task; PA: Physical Activity; WHO: World Health Organization.

**Table 3:** Multivariable binary logistic regression analysis of factors associated with meeting the WHO aerobic physical activity recommendations among patients with AxSpA (*n* = 153).

Variables	B	SE	*p*-Value	OR	95% CI
Age (years)	0.007	0.024	0.750	1.008	0.962–1.055
Sex (reference: male)	−1.295	0.569	**0.023**	0.274	0.090–0.835
FSS	−0.202	0.148	0.171	0.817	0.611–1.091
BASFI	−0.062	0.205	0.763	0.940	0.629–1.406
HAQ-S	−0.785	0.911	0.389	0.456	0.077–2.717
PSQI	0.051	0.067	0.454	1.052	0.922–1.200
ASAS-HI	0.110	0.098	0.261	1.116	0.922–1.351
TSK	−0.176	0.057	**0.002**	0.838	0.750–0.937

Abbreviations: B: Regression coefficient, OR: Odds ratio; CI: Confidence interval; SE: Standard error; FSS: Fatigue Severity Scale; BASFI: Bath Ankylosing Spondylitis Functional Index; HAQ-S: Health Assessment Questionnaire for Spondylarthritis; PSQI: Pittsburgh Sleep Quality Index; ASAS-HI: Assessment of Spondyloarthritis International Society Health Index; TSK: Tampa Scale for Kinesiophobia. Model statistics: The overall model was statistically significant (Omnibus χ^2^ = 32.398, df = 8, *p* < 0.001). Model calibration was adequate according to the Hosmer–Lemeshow goodness-of-fit test (χ^2^ = 3.213, df = 8, *p* = 0.920). Overall classification accuracy was 79.1%, with a sensitivity of 34.2% and specificity of 93.9%. Statistically significant associations are shown in bold (*p* < 0.05).

## Data Availability

The data presented in this study are available upon reasonable request from the corresponding author due to institutional privacy constraints.

## Supplementary Materials

The following supporting information can be downloaded at: https://www.mdpi.com/article/10.3390/jcm15176706/s1, Supplementary Materials S1: Exercise Preference and Barrier Questions Adapted from the Personalized Exercise Questionnaire (PEQ); Table S1: Univariate logistic regression analysis of variables potentially associated with meeting the WHO aerobic physical activity recommendations; Table S2: Sensitivity analysis of adherence to the WHO aerobic physical activity recommendations after excluding walking; Table S3: Responses to items adapted from the Personalized Exercise Questionnaire (PEQ).
