# Supplementary material for: Physical Activity, Guideline Compliance, and Exercise Preferences in Axial Spondyloarthritis: A Comparative Cross-Sectional Study
Source: J Clin Med. 2026 Aug 29;15(17):6706. doi: 10.3390/jcm15176706 (PMC13566743; doi:10.3390/jcm15176706)
Supplement: Supplementary file 1 [file jcm-15-06706-s001.zip › Supplementary Materials S1.pdf]

Supplementary Materials S1. Exercise Preference and Barrier Questionnaire

The following items were adapted from the Personalized Exercise Questionnaire and tailored to assess exercise preferences, perceived barriers, and support needs in participants.

1. Supervision or Assistance

1. I prefer someone to supervise or assist me during exercise.
Please select one option.

☐ Yes

☐ Not sure

☐ No

☐ Not applicable

If yes, whom would you prefer to be supervised by?
Please select all that apply.

☐ Healthcare professional

☐ Personal trainer

☐ Other: ____________

---

2. Preferred Exercise Setting

2. Where would you like your exercise program to take place?
Please select all that apply.

☐ Gym

☐ Home

☐ Outdoors

☐ Mall

☐ Community center

☐ Other: ____________

---

3. Preferred Exercise Time

3. What is the best time for you to exercise?
Please select all that apply.

☐ Morning, 06:00–12:00
☐ Afternoon, 12:00–18:00
☐ Evening, 18:00–23:00

---

4. Preferred Exercise Class Size

4. What is your preferred exercise class size?
Please select all that apply.

☐ I prefer to exercise alone
☐ With a partner or trainer
☐ Small group, <10 people
☐ Large group, >10 people
☐ Does not matter

---

5. Feedback on Exercise Progress

5. I would like to receive feedback about my exercise progress.
Please select one option.

☐ Yes
☐ No

If yes, how would you prefer to receive feedback?
Please select all that apply.

☐ In person
☐ Phone call
☐ Text message
☐ Email
☐ Social media

---

6. Type of Feedback

6. What type of feedback would you like to receive?
Please select all that apply.

☐ Feedback regarding my exercise progress and future improvements

☐ Feedback regarding proper exercise techniques

☐ Other: ____________

---

7. Frequency of Feedback

7. How often would you like to receive feedback about your exercise progress?
Please select one option.

☐ Daily

☐ Weekly

☐ Monthly

☐ Yearly

---

8. Providing Feedback on the Exercise Program

8. I would like to give feedback on the exercise program.
Please select one option.

☐ Yes

☐ No

If yes, how would you prefer to give feedback?
Please select all that apply.

☐ In person

☐ Phone call

☐ Text message

☐ Email

☐ Social media

---

9. Barriers to Exercise

9. I do not exercise as often as I would like because I do not have:
Please select all that apply.

☐ Time, for example family priorities, work, etc.

☐ Willpower or motivation

☐ Mobility, for example limited movement due to stiffness or pain

☐ A place to exercise
☐ Finances
☐ Proper quality of sleep
☐ Transportation
☐ Confidence, for example feeling self-conscious about my body
☐ Other: ____________
☐ None of the above

---

10. Weather Conditions

10. Do weather conditions stop you from exercising as often as you would like?
Please select one option.

☐ Always
☐ Very often
☐ Sometimes
☐ Rarely
☐ Never
