# Supplementary material for: Physical Activity, Guideline Compliance, and Exercise Preferences in Axial Spondyloarthritis: A Comparative Cross-Sectional Study
Source: J Clin Med. 2026 Aug 29;15(17):6706. doi: 10.3390/jcm15176706 (PMC13566743; doi:10.3390/jcm15176706)
Supplement: Supplementary file 1 [file jcm-15-06706-s001.zip › Supplementary Table S1.pdf]

Supplementary Table S1. Univariate logistic regression analysis of variables potentially associated with meeting the WHO aerobic physical activity recommendations

**Variable**	**B**	**SE**	**p**	**OR**	**95% CI**
Age	−0.021	0.019	0.285	0.979	0.943–1.017
Sex	−1.554	0.516	**0.003**	0.211	0.077-0.581
BMI	−0.042	0.038	0.274	0.959	0.890–1.033
PSQI total score	−0.063	0.049	0.196	0.939	0.853–1.033
FSS score	−0.393	0.118	<0.001	0.675	0.536–0.851
BASFI score	−0.309	0.134	0.021	0.734	0.564–0.955
HADS-D score	−0.029	0.045	0.515	0.971	0.889–1.061
HAQ-S total score	−1.413	0.526	0.007	0.243	0.087—0.682
ASQoL score	−0.130	0.043	0.003	0.878	0.807–0.955
ASAS-HI score	−0.121	0.051	0.018	0.886	0.802–0.980
TSK score	-0.190	0.047	<0.001	0.827	0.755–0.907

OR: odds ratio; **CI:** confidence interval; SE: standard error; **B**: regression coefficient; **BMI:** Body mass index, **PSQI**: Pittsburgh Sleep Quality Index, **FSS**: Fatigue Severity Scale; B**ASFI**: Bath Ankylosing Spondylitis Functional Index; **HADS-D**: Hospital Anxiety and Depression Scale–Depression subscale; **HAQ-S**: Health Assessment Questionnaire for Spondylarthritis; **ASQoL**: Ankylosing Spondylitis Quality of Life questionnaire; **ASAS-HI**: Assessment of Spondylarthritis International Society Health Index; **TSK**: Tampa Scale for Kinesiophobia.

Supplementary Table S2. Sensitivity analysis of adherence to the WHO aerobic physical activity recommendations after excluding walking

**Variable**	**B**	**SE**	**p**	**OR**	**95% CI**
AxSpA/Control group	−0.566	0.263	**0.032**	**0.568**	0.339–0.951
Age	−0.046	0.014	**0.001**	0.955	0.929–0.982
Sex	−0.744	0.294	**0.011**	0.475	0.267–0.846
BMI	−0.116	0.169	0.495	0.891	0.639–1.242

OR: odds ratio; **CI:** confidence interval; SE: standard error; **B**: regression coefficient; **AxSpA**: Axial spondylarthritis, **BMI:** Body mass index

Supplementary Table S3: Responses to items adapted from the Personalized Exercise Questionnaire (PEQ)

	AxSpA	Control	P	FDR-adjusted
**1. I prefer someone to supervise/assist me with an exercise:**			0.015	**0.015**
Yes	87(56.9)	73(47.7)		
Not sure	12(7.8)	17(11.1)		
No	53(34.6)	52(34.0)		
Not Applicable	1(0.7)	11(7.2)		
**If YES, by:**			<0.001	**<0.001**
Healthcare professional	54(62.1)	23(31.5)		
Personal trainer	28(32.2)	49(67.1)		
Other	5(5.7)	1(1.4)		
**2. Where would you like your exercise program to be? (Check all that apply)**				
Gym	76(49.7)	110(71.9)	<0.001	**0.004**
Home	77(50.3)	53(34.6)	0.006	**0.019**
Outdoors	62(40.5)	77(50.3)	0.085	0.134
Mall	2(1.3)	3(2.0)	1.000*	1.000
Community Centre	2(1.3)	4(2.6)	0.684*	0.717
Other	9(5.9)	3(2.0)	0.077	0.130
**3. What is the best time for you to exercise? (Check all that apply)**				
Morning (between 6 am and 12 pm)	57(37.3)	76(49.7)	0.028	0.056
Afternoon (between 12 pm and 6 pm)	31(20.3)	17(11.1)	0.028	0.056
Evening (between 6 pm and 11 pm)	77(50.3)	88(57.5)	0.207	0.268
**4. What is your preferred exercise session/group size?**				
I prefer to exercise alone	65(42.5)	77(50.3)	0.169	0.232
With a partner/trainer	54(35.3)	62(40.5)	0.346	0.423
Small group (less than 10 people)	22(14.4)	43(28.1)	0.003	**0.011**
Large group (more than 10 people)	6(3.9)	9(5.9)	0.427	0.494
Does not matter	30(19.6)	16(10.5)	0.025	**0.044**
**5. I would like to receive feedback about my progress:**				
Yes	105(68.6)	85(55.6)	0.018	**0.044**
No	48(31.4)	68(44.4)		
**If YES, by: (Check all that apply) (n=190)**				
In person	59(56.2)	52(61.2)	0.488	0.527
Phone call	49(46.7)	18(21.2)	<0.001	**0.004**
Text message	25(23.8)	10(11.8)	0.033	0.061
Email	13(12.4)	28(32.9)	0.001	**0.004**
Social media	9(8.6)	3(3.5)	0.155	0.227
**6. What type of feedback would you like to receive? (Check all that apply) (n=190)**				
Exercise progress and future improvements	80(76.2)	71(83.5)	0.213	0.645
Proper exercise techniques	73(69.5)	61(71.8)	0.736	0.743
Other	3(2.9)	1(1.2)	0.629	0.743

Supplementary Table S3: Responses to items adapted from the Personalized Exercise Questionnaire (PEQ) (continued)

	AxSpA	Control	P	FDR-adjusted
**7. How often would you like to receive feedback about your exercise progress? (Check all that apply) (n=116)**				
Daily	12(11.4)	6(7.1)	0.735	0.735
Weekly	52(49.5)	46(54.1)		
Monthly	36(34.3)	28(32.9)		
Yearly	5(4.8)	5(5.9)		
**8. I would like to give feedback on the exercise program:**				
Yes	91(59.5)	72(47.1)	**0.029**	**0.029**
No	62(40.5)	81(52.9)		
**If YES, by (Check all that apply): (n=163)**				
In person	50(54.9)	20(27.8)	0.001	**0.001**
Phone call	43(47.3)	18(25)	0.004	**0.003**
Text message	19(20.9)	9(12.5)	0.159	0.064
Email	9(9.9)	25(34.2)	<0.001	**0.001**
Social media	9(9.9)	2(2.8)	0.114	0.058
**9. I do not exercise as often as I like because I do not have: (Check all that apply)**				
Time (e.g., family priorities, work, etc.)	79(51.6)	91(59.5)	0.167	0.225
Willpower/motivation	60(39.2)	56(36.6)	0.637	0.515
Mobility (e.g., limited movements due to stiffness and pain)	66(43.1)	17(11.1)	<0.001	**0.004**
A place to exercise	38(24.8)	32(20.9)	0.414	0.397
Finances	32(20.9)	17(11.1)	0.019	**0.038**
Proper quality of sleep	34(22.2)	15(9.8)	0.003	**0.008**
Transportation	17(11.1)	13(8.5)	0.442	0.397
Confidence	23(15.0)	4(2.6)	<0.001	**0.004**
Other:	4(2.6)	12(7.8)	0.040	0.065
None of the above	23(15.0)	16(10.5)	0.230	0.265
**10. Do weather conditions stop you from exercising as often as you like?**			0.019	**0.019**
Always	4(2.6)	4(2.6)	1.000	1.000
Very often	20(13.1)	9(5.9)	0.032	0.080
Sometimes	50(32.7)	49(32.0)	0.903	1.000
Rarely	23(15.0)	44(28.8)	0.004	**0.020**
Never	56(36.6)	47(30.7)	0.276	0.460

Data are presented as n (%).
P values were calculated using the chi-square test or Fisher’s exact test, as appropriate, and FDR was performed. Significant p-values are presented in bold.
PEQ: Personalized Exercise Questionnaire; **AxSpA:** Axial spondylarthritis; **FDR**: False discovery rate
For each PEQ item, p-values corresponding to the respective response-category comparisons were adjusted separately using the Benjamini–Hochberg false discovery rate (FDR) procedure.
